# Gender sensitivity among general practitioners: Results of a training programme

**DOI:** 10.1186/1472-6920-8-36

**Published:** 2008-06-26

**Authors:** Halime H Celik, Ineke I Klinge, Trudy T van der Weijden, Guy GAM Widdershoven, Toine ALM Lagro-Janssen

**Affiliations:** 1 Department of Health, Ethics and Society, School for Public Health and Primary Care (CAPHRI), Faculty of Health Medicine and Life Sciences, Maastricht University, The Netherlands; 2 Department of General Practice/Centre for Quality of Care Research, School for Public Health and Primary Care (CAPHRI), Faculty of Health Medicine and Life Sciences, Maastricht University, The Netherlands; 3 Women's Studies Medical Sciences/General Practice, Radboud University Nijmegen, The Netherlands

## Abstract

**Background:**

Gender differences contribute to patients' health and illness. However in current healthcare practices attention to gender differences is still underdeveloped. Recognizing these differences and taking them into account can improve the quality of care. In this study we aimed to investigate whether GPs' gender sensitivity can be stimulated by a training programme. The focus was on three diseases: angina pectoris, depression and urinary incontinence.

**Methods:**

This study had a quantitative, explorative and descriptive design. By means of a training programme 18 GPs were trained to focus on gender-sensitive recommendations for the three diseases. With standardised registration forms, data were collected during a 6-month period. During the registration period, the GPs were visited by the study team to discuss the process of data collection.

**Results:**

The GPs filled in registration forms for 100 patients: 39 with angina pectoris (31 women and 8 men), 40 with depression (26 women and 14 men), and 21 with urinary incontinence (20 women and 1 man). The results show that gender sensitivity can be stimulated among trained professionals. The combination of the training programme, clear and practical recommendations, daily discussion of relevant cases between the GP couples, feedback and support during registration by the study team probably contributed to the outcome.

**Conclusion:**

GPs' gender sensitivity was stimulated by the training programme and the supporting visits. Ideally, structural attention could be realised by embedding gender issues in existing organisational structures of general practices.

## Background

Differences between men and women in health depend on the interaction of biological, psychological, socio-economic, and cultural factors [1-5]. Gender includes masculinity and femininity and refers to the array of socially constructed roles, behaviours and values that society ascribes to the two sexes on a differential basis. Recognition of gender differences can prevent inequity in caregiving. As a result the health of women and men can be improved [6,7]. In conventional medicine, a patient's illness is usually reduced to a set of signs and symptoms within a biomedical framework [8]. In this framework attention to gender issues is underdeveloped as evidenced by the clinical practice guidelines, which are widely used for decision-making in healthcare. To date, most guidelines do not cover gender issues [9]. Moreover, medical training and General Practitioner (GP) specialist training is gender neutral, as little or no attention is paid to sex and gender differences in medical education [10]. Therefore, it is not surprising that much about gender differences remains unknown and unaddressed in the provision of healthcare.

For adequate care the approach of health professionals should be more attuned to gender issues. Gender sensitivity of health professionals can be defined as showing sensitivity to gender issues in clinical decision-making. Optimizing this requires an awareness that gender (1) has an impact on health, and (2) affects the presentation of health complaints. This sensitivity allows health professionals to deal effectively with gender which will have positive effects on men as well as women.

We developed and implemented a training programme that focused on gender-sensitive recommendations. During the programme GPs were trained to take gender into consideration for three prevalent diseases in which gender is an important factor for the quality of care: angina pectoris, depression and urinary incontinence. The central question in this study is whether the training programme actually contributed to GPs' gender sensitivity.

## Methods

### Study design

A quantitative, explorative and descriptive research design was used as basis for our study. This study was part of a larger research project conducted by Maastricht University in collaboration with partners from the Academic Medical Centre of the University of Amsterdam and Radboud University Nijmegen Medical Centre. The study reported here, investigates the gender sensitivity of trained GPs in relation to suggested gender sensitive recommendations.

### Study population

The study population consisted of 9 experienced GPs (8 men and 1 woman) and 9 GPs in third year of training (2 men and 7 women). According to the standard training for general practitioners each GP trainee was coupled with another GP teacher, the academic instructor for the specialist training. They participated as volunteers in this study as a team from the same practice.

### Disease selection

Three important public health diseases were chosen: angina pectoris, depression, and urinary incontinence. These fulfilled the following selection criteria: availability of clinical practice guidelines, highly prevalent, and proven link with gender differences, the disease should be initially treated by GPs.

### Recommendations

A structured literature review using a previously described search strategy on the three diseases was performed by researchers at the University of Amsterdam, in scientific databases including Pubmed, Embase, and Psychinfo [9]. Articles were selected according to quality assessment (Cochrane), clinical relevance of gender aspects and presence of additional information compared to the existing guidelines of the Dutch College of General Practitioners (DCGP-guidelines). From the selected articles we formulated several recommendations with regard to these three diseases [11]. In this study we present these gender sensitive recommendations.

### Training programme

Based on the results of the literature review, an interactive training programme consisting of two modules was developed and executed by the coordinator of the programme (Toine Lagro-Janssen). For the programme description see [12]. The training programme focused on the recommendations (see boxes below) for a gender sensitive approach to the three diseases in the general practice. The modules included a general introduction in to gender-related issues and interactive lectures. Audio visual materials related to gender issues were used for discussion purposes with the participants by the coordinator of the programme. GPs were trained to put the recommendations into practice. The programme ended with instructions on how to use the designed registration forms containing the gender sensitive recommendations and how to discuss the contents for educational and research purposes.

#### Gender sensitive recommendations for angina pectoris

1. With chest pain attention should be paid to diabetes as a risk factor in women also when atypical symptoms for chest pain are present. The reason is that due to diabetes mellitus the risk for cardiovascular disease will increase double for men and triple for women [13].

2. Information about the socioeconomic status should be obtained, since the proportion of chest pain referring to angina pectoris increases with the decreasing level of socioeconomic status [14]. Furthermore, low socioeconomic status in women is related to higher morbidity from angina pectoris than for men [15].

3. Symptoms which do not immediately disappear in women during rest, should not be ignored, since there can be an underlying angina pectoris [16]. Typical symptoms for angina pectoris will present during physical activity and rapidly disappear with rest.

#### Gender sensitive recommendations for depression

1. The GP should ask whether there are sexual problems in depressed patients, since they do not often mention this spontaneously. In depressed patients there is generally a decrease of sexual functioning.

##### Specifically for women

1. GP's should know that depression in women can be masked by anxiety, since women with depression suffer from anxiety [17]. Furthermore, women report more symptoms consistent with anxiety than men [18].

2. GP's should ask depressed women about their past sexual experiences, since a history of sexual abuse is strongly associated with depression in women [19].

##### Specifically for men

1. The GP should know that depression in men could be masked by alcohol abuse [20], since depressed men show significantly more alcohol abuse [17,21].

2. In depressed male patients there is generally a decrease in libido, and erectile dysfunction. Men frequently experience loss of libido, and erectile dysfunction as a serious disability. The GP should ask whether the patient suffers from these symptoms, since men do not often mention this spontaneously [22].

#### Gender sensitive recommendations for urinary incontinence

1. Recommending the use of a diary in men and women can be an instrument for improving the quality of care.

2. GPs should consider sexual issues in the management of patients with incontinence, since incontinence is a risk factor for sexual dysfunction in men and women [23].

##### Specifically for women

1. GP's should promptly provide active treatment for women who consult their GP with incontinence which exists for a long period.

##### Specifically for men

1. The GP need to attend to the emotional well-being, since most men do not report distress spontaneously [24]. Men consulting a GP may not minimize the extent of their incontinence compared to women, due to their fear of prostate cancer or impotence. These underlying concerns may cause distress which negatively impacts on their well-being.

### Registrations

The GPs were asked to fill out standardised registration forms for patients seen for the first time for one of the three aforementioned diseases, immediately after the consultation. The purpose of the registration forms was to support and increase GPs' gender sensitivity toward patients. These quantitative data were collected during a 6-month period. Informed consent was obtained to use these data for scientific publications. If the GP did not follow a particular recommendation, the GP had to explain the reason for this on the form. At the end of the day all of the completed forms in the practice were discussed with each GP couple. The routine of daily conversations about their experiences and their difficult cases constituted a standard aspect of this gender- sensitivity training. During the 6-month period the study team visited the GP practices twice to discuss the process of data collection.

### Data analysis

To score gender sensitivity, all registration forms were coded. If GPs followed the recommendations, we assumed that they were more gender sensitive. Each recommendation was assigned a code 1 when the GP's adherence to the recommendation was gender sensitivity and 0 if the adherence was not gender sensitivity. Hence, not gender sensitive means non-adherence to the gender-sensitive recommendation. Take for example, the first recommendation for angina pectoris. In the event of chest pain attention should be paid to diabetes as a risk factor in women also when atypical symptoms are present. If attention was paid to diabetes as a risk factor in women with typical and atypical symptoms, the adherence was expected to be gender sensitive, and we assigned this result a code 1. If diabetes was not considered as a risk factor, this was not expected to be gender sensitive, and we gave this a code 0. Data were analysed with descriptive statistics, using SPSS 13.

## Results

In total 100 registration forms for patients were completed (39 patients with angina pectoris, 40 patients with depression, and 21 patients with urinary incontinence).

### Angina Pectoris

Of 39 patients with angina pectoris, GPs registered 31 female and 8 male patients. As shown in Table 1, diabetes was considered for almost all female and male patients. In the GP practices references to socioeconomic status (financial problems) and complaints of chest pain during rest periods were relatively more frequent noticed for men than for women. Reasons given for not determining the socioeconomic status for male patients were the urgency of the medical situation and difficulty to apply this principle. In addition to the urgency of the medical situation, reasons given for not determining the socioeconomic status of women were: prior knowledge of this (N = 1), forgetting, or considering the symptoms as unproblematic.

**Table 1:** Indicators for angina pectoris

**Angina Pectoris N = 39**		
N = 31 female; N = 8 male		
*Indicators*	Considered in women	Considered in men

***Diabetes***	30 (97%)	8 (100%)
***Socioeconomic status***	10 (34%)	4 (50%)
***Complaints during rest***	18 (58%)	6 (75%)

### Depression

Registration forms were filled out for 40 patients with depression (26 women and 14 men). For female patients suffering form depression, the GPs acted according to the recommendation regarding anxiety in the case of 25 patients (96%) (Table 2). The recommendation regarding sexual abuse was followed for 8 female patients (31%). Reasons given for not asking depressed women about their sexual history were: the GP did not find an appropriate opportunity to ask about it, it was difficult to ask during the first consultation due to the sensitive nature of the question, the patients related their sexual history spontaneously, or the GP already knew the sexual history. Sexual problems were addressed in the case of 15 (58%) female patients. Alcohol abuse was discussed with all male patients, while sexual problems in men were discussed with 4 (29%) patients (Table 3).

**Table 2:** Indicators for women with depression

**Depression N = 26**	
*Indicators*	Considered

***Anxiety***	25 (96%)
***Sexual abuse***	8 (31%)
***Sexual problems***	15 (58%)

**Table 3:** Indicators for men with depression

**Depression N = 14**	
*Indicators*	Considered

***Alcohol abuse***	14 (100%)
***Sexual problems***	4 (29%)

### Urinary incontinence

Of 21 patients with urinary incontinence were 20 women and 1 man registered. The results suggest that the presentation of urinary incontinence complaints to the GP was low, particularly among male patients. During the registration period GPs reported that this could be because patients do not schedule a consultation, but just ask the GP assistant by phone to prescribe incontinence pads for them. The recommendation to advise the patient to keep a diary for urinary incontinence was followed by the GPs for 2 (11%) of the female patients. The recommendation for active treatment and addressing sexual dysfunction was followed for 15 (79%) and 11 (58%) of the female patients, respectively, as shown in Table 4. Since, there was only one male patient; the result was not included.

**Table 4:** Indicators for women with urinary incontinence

**Incontinence **N = 20 female	
*Indicators*	Considered

***Diary keeping***	2 (11%)
***Active treatment***	15 (79%)
***Sexual dysfunction***	11 (58%)

### Gender sensitivity

GPs mean gender sensitivity for all patients was 1.84 (SD:.79). That means that on average, the GPs applied two out of three recommendations to all patients.

## Discussion

To our knowledge this is the first study that investigates gender sensitivity of GPs which extends beyond the existing guidelines. The results show that gender sensitivity can be stimulated among trained professionals for non-routine patient cases. The combination of the training programme, clear and practical recommendations, daily discussion of relevant cases by the GP couples, as well as feedback and support from the study team probably all contributed to the outcome.

During the registration period, the GPs were visited by the study team to discuss the process of data collection. These visits served as a reminder to fill out the registration forms and were aimed at working out the practical difficulties of applying the recommendations in the GP practice. During the visits we found that GP teams did not fill out a form for all first visits of patients suffering from angina pectoris, depression and urinary incontinence. Interestingly, the GPs did not register the routine cases, but selected patients with expected gender-related problems to register and discuss with each other for educational purposes.

Our data show that more registration forms were completed for female than for male patients for the three conditions. An explanation could be that women have more contact with their GPs than men [25,26]. For angina pectoris in particular special attention to women was deemed urgently warranted during the training programme. This was in reaction to the tendency to the focus on typical symptoms and a masculine presentation style [27]. This focus was not to minimize the gender effects on the health of men with angina pectoris, but rather to correct existing imbalances in the general practice. Even though attention should be paid to atypical symptoms in women, attention to typical symptoms is still required for both sexes. Concerning depression and urinary incontinence, the number of registration forms completed for female patients can be explained by higher prevalence of these conditions in women in the general practice [28]. In line with earlier studies, it is also possible that an increasing awareness of gender issues in medical education and practice has resulted in an increased focus on women [29].

In general, GPs adherence to the gender sensitive recommendations in this study was relatively high. We did not find differences in gender sensitivity towards women and men. Recommendations regarding the socioeconomic status of patients with angina pectoris, sexual abuse in women with depression, sexual problems in men with depression, and diary keeping in women with urinary incontinence, were followed less frequently. In instances when the GP already knew the socioeconomic status or the patient related the sexual history spontaneously, these recommendations were obviously irrelevant. Nevertheless, in the majority of the cases the reasons for not following these recommendations -e.g. forgetting to ask or reluctance to discuss sexual history during first consultation- leave room for improvement.

This study can be regarded exploratory in character. To determine the effects of the programme a study with before-after measurement and/or a concurrent control group is necessary. The effect of the training programme may have been boosted by the practice visits during the study period. The samples per professional were too small to detect a trend in the GPs' sensitivity over the 6-months period. For a large-scale and long-term effect, it should be kept in mind that a training programme for general practitioners is just one of the sources to improve the position of gender issues in medical decision-making. Ideally, progress could be realised by embedding gender issues into existing organisational structures of the general practice. This is in agreement with findings in our earlier study [30]. Organizational constraints can be a barrier to incorporating and maintaining gender issues into healthcare practices. Therefore it is important to explore ways of effectively addressing gender issues in the organisation of the family practice by developing tailored recommendations. Further qualitative research of GPs' impressions and feedback on this subject is recommended as it will complement gender-related research and practice.

## Competing interests

The authors declare that they have no competing interests.

## Authors' contributions

**HHC: **substantial contributions to research design, the acquisition, analysis and interpretation of data; drafting the paper; approval of the submitted and final versions.** IIK: **substantial contributions to the acquisition of data; revising the paper critically; approval of the submitted and final versions.** TvdW: **substantial contributions to the interpretation of data; revising the paper critically; approval of the submitted and final versions.** GGAMW**: substantial contributions to the interpretation of data; revising the paper critically; approval of the submitted and final versions.** TALML-J**: substantial contributions to research design, the acquisition, and interpretation of data; drafting the paper and revising it critically; approval of the submitted and final versions.

## Pre-publication history

The pre-publication history for this paper can be accessed here:


